# Dynamics of the fecal microbiome and antimicrobial resistome in commercial piglets during the weaning period

**DOI:** 10.1038/s41598-021-97586-9

**Published:** 2021-09-10

**Authors:** Prapat Suriyaphol, Jimmy Ka Ho Chiu, Nathamon Yimpring, Paiboon Tunsagool, Wuttichai Mhuantong, Rungtip Chuanchuen, Irina Bessarab, Rohan B. H. Williams, Rick Twee-Hee Ong, Gunnaporn Suriyaphol

**Affiliations:** 1 Office for Research and Development, Faculty of Medicine, Siriraj Hospital, Mahidol University, Bangkok, 10700 Thailand; 2 Saw Swee Hock School of Public Health, National University of Singapore and National University Health System, Tahir Foundation Building National University of Singapore, 12 Science Drive 2, #10-01, Singapore, 117549 Singapore; 3 Biochemistry Unit, Department of Physiology, Faculty of Veterinary Science, Chulalongkorn University, 39, Henri Dunant Road, Wangmai, Pathumwan, Bangkok, 10330 Thailand; 4 Department of Biotechnology, Faculty of Agro-Industry, Kasetsart University, Bangkok, 10900 Thailand; 5 Biorefinery and Bioproduct Technology Research Group, National Center for Genetic Engineering and Biotechnology, National Science and Technology Development Agency, Pathum Thani, 12120 Thailand; 6 Research Unit in Microbial Food Safety and Antimicrobial Resistance, Department of Veterinary Public Health, Faculty of Veterinary Science, Chulalongkorn University, Bangkok, 10330 Thailand; 7 Singapore Centre on Environmental Life Sciences Engineering (SCELSE), Nanyang Technological University, Singapore, 639798 Singapore

**Keywords:** Microbiology, Molecular biology, Systems biology

## Abstract

This study aimed to characterize the alteration of the fecal microbiome and antimicrobial resistance (AMR) determinants in 24 piglets at day 3 pre-weaning (D. − 3), weaning day (D.0), days 3 (D.3) and 8 post-weaning (D.8), using whole-genome shotgun sequencing. Distinct clusters of microbiomes and AMR determinants were observed at D.8 when *Prevotella* (20.9%) was the major genus, whereas at D. − 3–D.3, *Alistipes* (6.9–12.7%) and *Bacteroides* (5.2–8.5%) were the major genera. *Lactobacillus* and *Escherichia* were notably observed at D. − 3 (1.2%) and D. − 3–D.3 (0.2–0.4%), respectively. For AMR, a distinct cluster of AMR determinants was observed at D.8, mainly conferring resistance to macrolide–lincosamide–streptogramin (*mef*A), β-lactam (*cfx*A6 and *aci*1) and phenicol (*rlm*N). In contrast, at D. − 3–D.3, a high abundance of determinants with aminoglycoside (AMG) (*sat*, *aac*(6')*-aph*(2''), *aad*A and *acr*F), β-lactam (*fus*-1, *cep*A and *mrd*A), multidrug resistance (MDR) (*gad*W, *mdt*E*, emr*A, *evg*S*, tol*C and *mdt*B), phenicol (*cat*B4 and *cml*A4), and sulfonamide patterns (*sul*3) was observed. Canonical correlation analysis (CCA) plot associated *Escherichia coli* with *aac*(6')*-aph*(2''), *emr*A, *mdt*B, *cat*B4 and *cml*A4 at D. − 3, D.0 and/or D.3 whereas at D.8 associations between *Prevotella* and *mef*A, *cfx*A6 and *aci*1 were identified. The weaning age and diet factor played an important role in the microbial community composition.

## Introduction

During the weaning period, piglets face several stress factors owing to either physiological changes or farm management. Physiological changes include (1) loss of protective sow’s milk immune cells, lactoferrin and lysozyme, (2) the presence of probiotic lactic acid bacteria, and (3) the shrinkage of villi in the small intestine, which reduces nutrient digestion and absorption^1–3^. The new environment due to farm management includes being transferred from a farrowing pen to an early-weaning unit, being mixed with unfamiliar pigs, and feed changes from highly digestible milk protein and sugars to creep feed and complex solid feed^4^. All of these factors can impair immune functions and lead to post-weaning diarrhoea syndrome (PWDS), resulting in diarrhoea, dehydration, depression, inappetence and weight loss. PWDS is usually associated with enterotoxigenic *Escherichia coli* strains (ETEC)^5^. The severe enteric infections might lead to the overuse of antibiotics. Imbalance of the normal gut microbiota in pigs has been reported to be associated with enteric diseases such as PWD and with the flare up of pathogens even not in the gastrointestinal tract system such as *Mycoplasma hyopneumoniae*^6,7^. The enhancement of gut microbiome ability to increase disease resistance in pigs and reduce antibiotic use are the major challenges in pig industry. Several nonantibiotic alternatives have been applied to decrease gut microbiota dysbiosis in pig industry such as zinc supplements, essential oils, short- and medium-chain organic acids, prebiotics and probiotics^8–11^.

In fact, several probiotic microorganisms are commonly used to inhibit bacterial infection such as *Lactobacillus plantarum*, *Bacillus licheniformis* and *Bacillus subtilis*. *L. platarum*, normal flora in porcine gastrointestinal tracts, has been reported to induce endogenous antimicrobial peptide synthesis in weaning piglets and improve pig growth and pork quality^12,13^. Pigs that received a probiotic-containing *Bacillus licheniformis* were shown to reduce porcine epidemic diarrhea virus infected vero cells^14^. In addition, a probiotic‐containing *Bacillus licheniformis* and *Bacillus subtilis* spores could improve productive performance and carcass quality in pigs^15^. However, probiotic microorganisms such as *Enterococcus faecium* and *B. subtilis* might serve as a reservoir for antimicrobial resistance (AMR) determinants^16,17^. Subtherapeutic antibiotics have been widely used to improve growth performance and food efficiency of weaned piglets. Low-dose chlortetracycline (0.5–1 g/sow daily) was used to increase breeding, conception rate, farrowing rates and litter size in sows^18^. Nonetheless, antimicrobial growth promoters can promote AMR strains of both Gram-positive and Gram-negative bacteria^19^. The utilization of antibiotics as growth promoters has been totally banned in the European Union since January 1, 2006^20^. In Thailand, utilization of antibiotics as growth promoters in food animals is banned^21,22^. In fact, antimicrobial prophylaxis at therapeutic doses has been generally used on commercial farms for a short time period to treat and prevent bacterial infection during parturition and weaning. These chemicals might cause antibiotic selective pressure and lead to bacterial genome evolution, allowing AMR variants to survive and multiply and disturbing gut microbial balance^23^. AMR of several antimicrobials has been reported in weaned pigs (from 0 to 28 days post-weaning), receiving chlortetracycline at a therapeutic dose and penicillin at a metaphylactic dose^24^. Resistant bacteria play an important role not only in pig health management but also in human health as AMR could be passed to human via direct contact or as a consumer^25^. Since environment can also be a reservoir for AMR resistome and maintain AMR genes, the ‘One Health’ approach to AMR surveillance in humans, animals and environments is required, not only at the laboratory level but also at the epidemiological, clinical and population levels^26^.

The objective of this study is to characterize the effect of weaning on the alteration of gut microbiome communities and AMR determinants in 24 commercial piglets (Landrace × Large White × Duroc Jersey) at day 3 pre-weaning (D. − 3), weaning day (D.0), days 3 (D.3) and 8 post-weaning (D.8) from 3 sows, using whole-genome shotgun sequencing.

## Results

### Microbiome diversity and abundance

**Figure 1:**
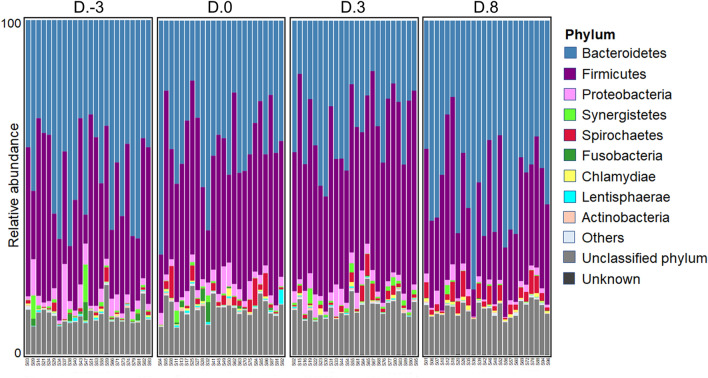
Stacked bar plot showing taxonomic relative abundance distribution of fecal microbial communities at the phylum level of piglets, with samples sub-categorized by maternal litter and age at day 3 pre-weaning (D. − 3), weaning day (D.0), days 3 (D.3) and 8 post-weaning (D.8).

**Figure 2:**
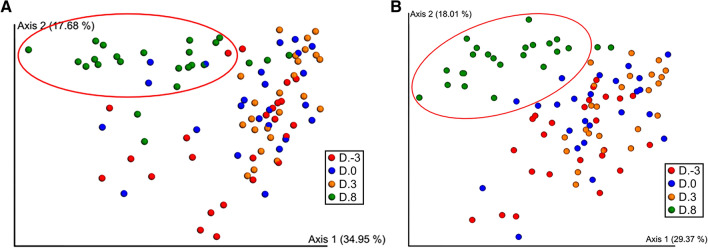
Principal coordinates analysis (PCoA) plot of antimicrobial resistance determinants obtained from piglets at day 3 pre-weaning (D. − 3), weaning day (D.0), days 3 (D.3) and 8 post-weaning (D.8). The oval shows a D.8 sample group that is separated from. D. − 3–D.3 groups. Taxonomic abundance (**A**). AMR gene abundance (**B**). Beta diversity group significance: *P* value = 0.001, PERMANOVA, 999 permutations.

The Shannon diversity index showed no statistical difference of the microbiomes across weaning ages (Supplementary Fig. S1). Across the entire cohort of samples, the relative abundance of *Bacteroidetes* and *Firmicutes* constituted approximately 80% of all bacteria phyla (Fig. 1, Table 1). Bacterial beta diversity demonstrated a major shift in the gut bacterial community during suckling–weaning transition. PCoA of taxonomy demonstrated that community composition on D.8 (post-weaning) differed from that of the other sampling days (Fig. 2A). Adonis confirmed that the community composition was predominantly associated with weaning age and diet (*P* < 0.01) (Table 2). Relative abundance at the genus level is shown in Table 3 and Supplementary Table S1. *Alistipes* was markedly high at D. − 3, whereas *Bacteroides* and *Parabacteroides* were prominently enriched at D. − 3–D.3 (*P* < 0.05). *Prevotella* and *Treponema* were more abundant at D.8 compared with D. − 3–D.3 (*P* < 0.05).

**Table 1:** The relative abundance of phyla in piglets at day 3 pre-weaning (D. − 3), weaning day (D.0), days 3 (D.3) and 8 post-weaning (D.8).

Phyla	Relative frequency (%) ± SD			
	D. − 3	D.0	D.3	D.8
*Bacteroidetes*	46.01 ± 13.60^ab^	37.88 ± 12.72^ac^	31.72 ± 11.22^c^	49.73 ± 14.34^b^
*Firmicutes*	35.38 ± 13.77^ab^	40.37 ± 11.77^ac^	47.20 ± 8.90^c^	30.46 ± 10.60^b^
*Proteobacteria*	3.63 ± 3.60^a^	3.08 ± 1.73^a^	3.12 ± 2.23^a^	1.54 ± 1.12^b^
*Synergistetes*	1.22 ± 2.52^ab^	0.47 ± 0.77^a^	0.98 ± 1.02^b^	0.21 ± 0.55^c^
*Spirochaetes*	0.62 ± 0.97^a^	2.16 ± 2.66^b^	1.54 ± 1.70^b^	3.07 ± 2.12^c^
*Fusobacteria*	0.57 ± 1.28^ab^	0.61 ± 1.39^a^	0.06 ± 0.06^a^	0.03 ± 0.04^b^
*Chlamydiae*	0.25 ± 0.23^a^	0.40 ± 0.49^a^	0.34 ± 0.43^a^	0.58 ± 0.33^b^
*Lentisphaerae*	0.24 ± 0.22^a^	0.44 ± 0.83^a^	0.19 ± 0.15^a^	0.04 ± 0.05^b^
*Actinobacteria*	0.19 ± 0.15^a^	0.32 ± 0.42^a^	0.27 ± 0.22^b^	0.20 ± 0.07^a^
Others	0.18 ± 0.02^a^	0.22 ± 0.02^ab^	0.24 ± 0.02^ab^	0.34 ± 0.08^b^
Unclassified phylum	11.52 ± 2.90^a^	13.85 ± 3.06^b^	14.16 ± 3.11^b^	13.52 ± 2.10^b^
Unknown	0.20 ± 0.07^a^	0.18 ± 0.06^a^	0.18 ± 0.06^a^	0.26 ± 0.05^b^

^a,b,c^A significant difference in the same row at *P* < 0.05.

**Table 2:** Adonis results for gut microbiome in piglets from day 3 pre-weaning to day 8 post-weaning.

Groups	R^2^-distances	*P* values
Weaning age and diet	0.243	0.001
Litter	0.035	0.058
Piglet	0.187	0.998

**Table 3:** Relative abundances of the 10 most abundant genera in each piglet group at day 3 pre-weaning (D. − 3), weaning day (D.0), days 3 (D.3) and 8 post-weaning (D.8).

Taxonomy	Relative frequency (%) ± SD			
	D. − 3	D.0	D.3	D.8
*Alistipes*	12.69 ± 9.99^a^	6.86 ± 4.90^ab^	7.04 ± 7.30^b^	6.01 ± 6.13^b^
*Bacteroides*	8.54 ± 6.66^a^	8.23 ± 5.68^a^	5.22 ± 2.97^a^	2.73 ± 0.79^b^
*Prevotella*	7.18 ± 7.32^a^	6.96 ± 6.31^a^	3.63 ± 2.00^a^	20.88 ± 11.28^b^
*Clostridium*	4.68 ± 4.51^ab^	7.98 ± 5.38^a^	6.84 ± 5.05^a^	2.84 ± 2.21^b^
*Parabacteroides*	1.20 ± 0.70^a^	1.20 ± 0.80^a^	2.73 ± 3.08^b^	0.37 ± 0.55^c^
*Lactobacillus*	1.17 ± 1.69^a^	0.22 ± 0.18^b^	0.34 ± 0.44^b^	0.96 ± 2.04^ab^
*Eubacterium*	0.84 ± 0.91^a^	0.48 ± 0.44^ab^	0.34 ± 0.30^b^	0.63 ± 0.33^a^
*Pyramidobacter*	0.83 ± 2.48^a^	0.25 ± 0.73^a^	0.13 ± 0.13^a^	0.02 ± 0.05^b^
*Butyricimonas*	0.81 ± 0.65^ab^	1.17 ± 1.40^a^	0.43 ± 0.33^b^	0.10 ± 0.08^c^
*Phascolarctobacterium*	0.57 ± 0.42^a^	0.73 ± 1.01^a^	0.26 ± 0.23^b^	0.61 ± 0.27^a^
*Treponema*	0.49 ± 0.78^a^	1.67 ± 2.42^b^	1.16 ± 1.32^ab^	2.64 ± 1.95^c^
*Fusobacterium*	0.48 ± 1.07^ab^	0.49 ± 1.09^a^	0.04 ± 0.05^a^	0.02 ± 0.04^b^
*Oscillibacter*	0.47 ± 0.33^a^	0.66 ± 0.39^ab^	0.54 ± 0.20^a^	0.82 ± 0.33^b^
*Desulfovibrio*	0.37 ± 0.24^a^	0.33 ± 0.17^a^	0.55 ± 0.34^a^	0.10 ± 0.08^b^
*Faecalibacterium*	0.23 ± 0.28^a^	0.26 ± 0.18^ab^	0.43 ± 0.48^b^	0.61 ± 0.36^c^

^a,b,c^A significant difference in the same row at *P* < 0.05.

### AMR determinant diversity and abundance

The top three abundant antimicrobial drug resistance patterns were aminoglycoside (AMG), tetracycline and macrolide-lincosamide-streptogramin (MLS), followed by multidrug resistance (MDR), β-lactam and phenicols (Table 4). D.8 showed a distinct PCoA cluster from other ages (Fig. 2B). A high abundance of AMR determinants observed on at least 2 days during D. − 3–D.3 conferred resistance to AMG, including *sat*, *aac*(6′)*-aph*(2″), *aad*A and *acr*F, resistance to β-lactam, including *fus*-1 (*oxa*-85), *cep*A and *mrd*A, resistance to MDR, including *gad*W, *mdt*E*, emr*A, *evg*S*, tol*C and *mdt*B, resistance to phenicol, including *cat*B4 and *cml*A4, and resistance to sulfonamide, including *sul*3. In contrast, a higher abundance of AMR determinants conferring resistance to MLS, *mef*A, resistance to β-lactam, *cfx*A6 and *aci*1, and resistance to phenicols, *rlm*N, was observed at D.8 (Supplementary Table S2).

**Table 4:** Percent of fragments per kilobase of exon model per million reads mapped (FPKM) of antimicrobial drug resistance patterns in piglets at day 3 pre-weaning (D. − 3), weaning day (D.0), days 3 (D.3) and 8 post-weaning (D.8).

Antimicrobial drug resistance patterns	Relative frequency (%) ± SD			
	D. − 3	D.0	D.3	D.8
Aminoglycosides	33.33 ± 7.07^a^	36.37 ± 7.71^ab^	39.47 ± 5.48^b^	25.56 ± 7.46^c^
Tetracyclines	29.89 ± 7.03^ab^	28.77 ± 4.39 ^ab^	28.74 ± 6.37^a^	30.94 ± 2.53^b^
Macrolide-Lincosamide-Streptogramin (MLS)	19.97 ± 4.28^a^	20.77 ± 4.64^a^	19.61 ± 4.21^a^	30.12 ± 6.02^b^
Multidrug resistance (MDR)	5.58 ± 5.54^a^	3.86 ± 3.45^a^	3.76 ± 4.15^a^	0.84 ± 1.37^b^
β-lactams	4.22 ± 2.71^a^	4.32 ± 2.44^a^	2.37 ± 1.05^b^	6.31 ± 2.73^c^
Phenicols	3.33 ± 1.88^a^	3.36 ± 1.29^a^	3.73 ± 1.49^a^	5.05 ± 1.65^b^
Sulfonamides	0.57 ± 0.56^a^	0.46 ± 0.47^ab^	0.23 ± 0.24^b^	0.07 ± 0.21^c^
Others	3.10 ± 1.82	2.09 ± 0.96	2.09 ± 1.21	1.11 ± 0.76

^a,b,c^A significant difference in the same row at *P* < 0.05.

### Inter-relationships between abundance profiles of member taxa and AMR sequence

**Figure 3:**
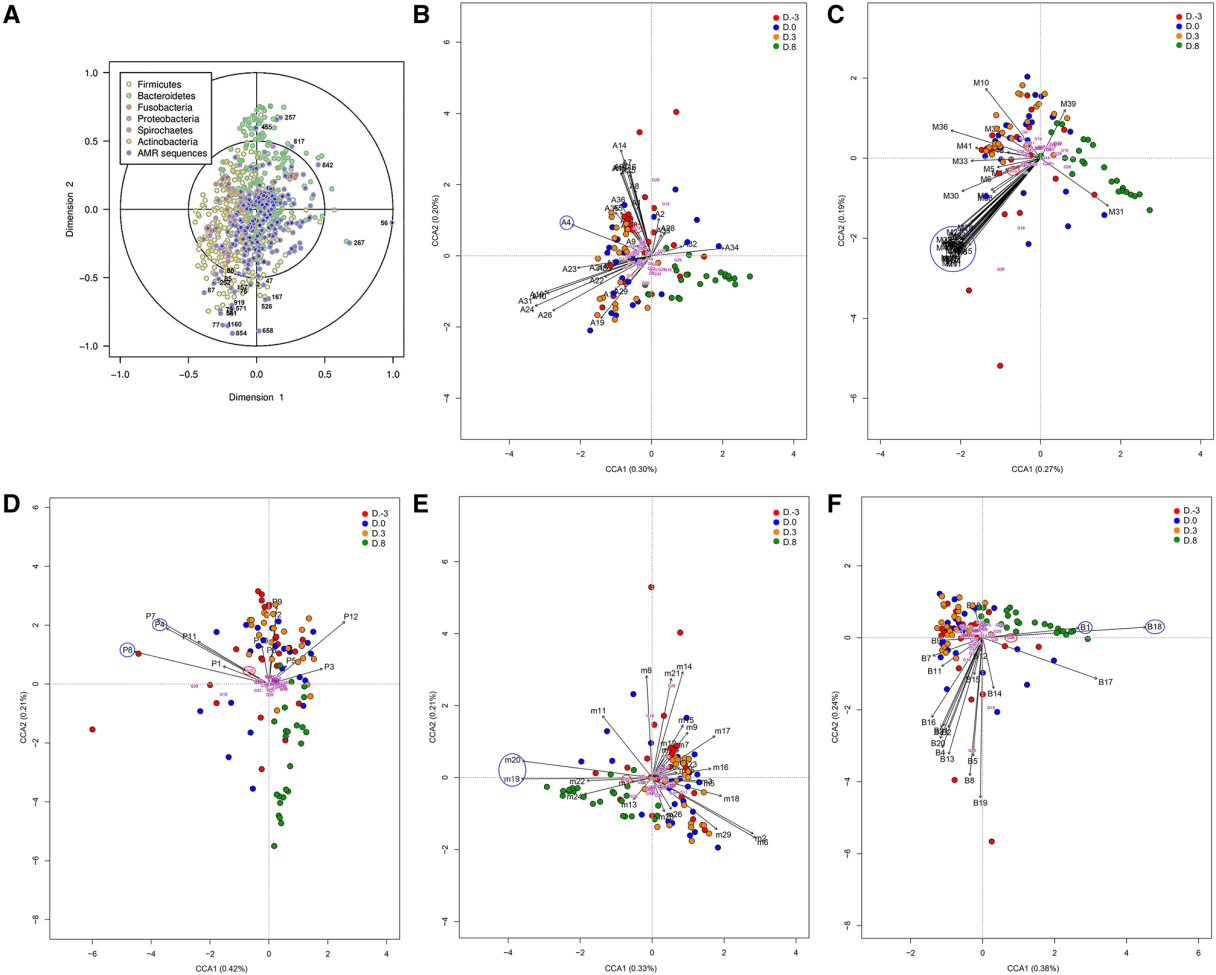
Canonical correlation analysis (CCA) plot showing the distribution of piglets at day 3 pre-weaning (D. − 3), weaning day (D.0), days 3 (D.3) and 8 post-weaning (D.8). Microbiome genera and antimicrobial resistance (AMR) genes according to corresponding antimicrobial drug resistance patterns. CCA plot showing inter-relationships between taxon abundance profiles and AMR sequences over all 96 samples (**A**). CCA plot of AMR genes corresponding to aminoglycoside drug resistance pattern (**B**). CCA plot of AMR genes corresponding to multidrug resistance pattern (**C**). CCA plot of AMR genes corresponding to phenicol drug resistance patterns (**D**). CCA plot of AMR genes corresponding to macrolide, lincosamide and streptogramin resistance (MLS) patterns (**E**). CCA plot of AMR genes corresponding to β-lactam resistance patterns (**F**). Blue circles indicate high abundant genes correlated with *Escherichia* or *Prevotella*. Red circles indicate *Escherichia* (G13) and *Prevotella* (G26). AMR genes (**A**) *rpo*B2 (2), *par*YR (47), *tet*(Q) (56), *tet*(32) (75), *tet*(O/W) (77), *tet*(W/32/O) (79), *tet*(W) (85), tet(O/W/O) (86), *tet*(W/N/W) (87), ABC transporter gene (157), *aac*(6')*-aph*(2'') (167), *tet*(40) (252), *mef*A (257), *mef*(En2) (267), *cfx*A6 (455), *aad*(6) (541), bifunctional aminoglycoside modifying enzyme gene (571), hygromycin-B kinase gene (526), *aad*E (658), *isa*E or putative spectomycin adenyltransferase gene (817), *erm*F (842), *aph*A3 (857), *dam* (919), *sat*4 (1160); Genus: *Alistipes* (G1), *Anaeromassilibacillus* (G2), *Anaerotruncus* (G3), *Bacteroides* (G4), *Blautia* (G5), *Butyricicoccus* (G6), *Butyricimonas* (G7), *Chlamydia* (G8), *Cloacibacillus* (G9), *Clostridium* (G10), *Culturomica* (G11), *Desulfovibrio* (G12), *Escherichia* (G13), *Eubacterium* (G14), *Faecalibacterium* (G15), *Fibrobacter* (G16), *Flavonifractor* (G17), *Fusobacterium* (G18), *Intestinimonas* (G19), *Lachnoclostridium* (G20), *Lactobacillus* (G21), *Odoribacter* (G22), *Oscillibacter* (G23), *Parabacteroides* (G24), *Phascolarctobacterium* (G25), *Prevotella* (G26), *Pseudoflavonifractor* (G27), *Pyramidobacter* (G28), *Roseburia* (G29), *Sphaerochaeta* (G30), *Streptococcus* (G31), *Subdoligranulum* (G32), *Sutterella* (G33), *Treponema* (G34), *Veillonella* (G35); Aminoglycoside resistance genes (**B**) *aac*(3) (A1), *aac*(6) (A2), *aac*(6) (A3), *aac*(6')*-aph*(2'') (A4), *aac*A4 (A5), *aac*C4 (A6), *aad* (A7), *aad*A15 (A8), *aad*A5 (A9), *aad*E (A10), *aad*E (A11), *aad*E (A12), *aad*K (A13), *aad*S (A14), *acr*D (A15), *acr*E (A16), *acr*F (A17), *ant*(6) (A18), *ant*(6) (A19), *ant*(9) (A20), *aph*(2) (A21), aph(2) (A22), *aph*(2) (A23), *aph*(3) (A24), *env*R (A25), *hph* (A26), *kdp*E (A27), *neo* (A28), *npm*A (A29), *rmt*F (A30), *sat* (A31), *sat* (A32), *sph* (A33), *spw* (A34), *str*A (A35), *str*B (A36); Multidrug resistance genes (**C**): *acr*A (M1), *acr*B (M2), *bae*S (M3), *cme*A (M4), *cme*B (M5), *cme*C (M6), *cpx*A (M7), *crp* (M8), *EB* (M9), *efr*A (M10), *emr*A (M11), *emr*B (M12), *emr*K (M13), *emr*Y (M14), *evg*A (M15), *evg*S (M16), *gad*W (M17), *hns* (M18), *mar*A (M19), *mdf*A (M20), *mdt*A (M21), *mdt*B (M22), *mdt*C (M23), *mdt*E (M24), *mdt*F (M25), *mdt*G (M26), *mdt*H (M27), *mdt*K (M28), *mdt*M (M29), *mef*(En2) (M30), *mef*G (M31), *mel* (M32), *mex*F (M33), *mpr*A (M34), *msb*A (M35), *oqx*B (M36), *qac*H (M37), *sme*B (M38), *sme*R (M39), *tae*A (M40), *tlr*C (M41), *tol*C (M42), *yjc*P (M43), *yjc*Q (M44), *yjc*R (M45), *yoj*I (M46); Phenicol resistance genes (**D**): *cat*B4 (P4), *cml* (P7), *cml*A4 (P8), *rlm*N (P12); MLS resistance genes (**E**) *ABC* (m1), *ABC* (m2), *ere*D (m3), *erm* (m4), *erm* (m5), *erm*2 (m6), *erm*33 (m7), *erm*35 (m8), *erm*47 (m9), *erm*A (m10), *erm*FS (m11), *erm*M (m12), *erm*Q (m13), *lin*G (m14), *lnu*B (m15), *lnu*C (m16), *lsa*E (m17), *mac*B (m18), *mef*A (m19), *mef*A (m20), *mef*B (m21), *mef*E (m22), *mph*A (m23), *mph*B (m24), *mph*E (m25), *ole*B (m26), *sat*G (m27), *srm*B (m28), *tlr*C (m29); Beta-lactam resistance genes (**F**) *aci*1 (B1), *amp*C (B2), *amp*H (B3), *bla* (B4), *bla* (B5), *bla* (B6), *bla* (B7), *bla*CARB (B8), *bla*LAP (B9), *bla*OXA (B10), *bla*OXA (B11), *bla*OXA (B12), *bla*TEM (B13), *bla*VEB (B14), *cbl*A (B15), *cep*A (B16), *cfx*A5 (B17), *cfx*A6 (B18), *fus*-1 (B19), *mrd*A (B20), *omp*K37 (B21).

According to the CCA plots, highly abundant AMR genes possibly correlated with *E. coli* on at least 2 days during D. − 3–D.3 compared with D.8, including AMG (*aac*(6')*-aph*(2'')), MDR (*emr*A and *mdt*B), and phenicol (*cat*4B *and cml*A4) resistance patterns. In contrast, AMR gene abundances possibly correlated with *Prevotella* at D.8 were in MLS (*mef*A) and β-lactam (*cfx*A6 and *aci*1) resistance patterns (Fig. 3, Supplementary Table S3).

## Discussion

This study revealed the population of fecal microbes and AMR determinants in commercial piglets aged 21–32 days. The Shannon diversity index is used to measure species richness and evenness. Divergent data of alpha diversity have been reported in piglets during weaning period. Increased indices were demonstrated in pigs at 10 and 21 days after weaning, compared with the weaning day and 10 days before the weaning day, showing the mature gut microbiome^27^. However, early weaning could lead to decreased alpha diversity, possibly due to weaning stress^28^. In our study, the weaning day was the same as Chen et al.^27^, however, we did not find significant differences in the Shannon diversity index among groups (days). This is possibly due to different feeding schedules, breeds and farm environments between the studies, and other factors may also affect the alpha diversity changes. However, we found that the microbial community of piglets at D.8 significantly differed from earlier sampling dates, probably as the consequence of physiological changes and post-weaning farm management practices. Moreover, the PERMANOVA results revealed that the weaning age and diet played an important role in gut microbiome populations (Table 2). *Alistipes* was prominently present at D. − 3 followed by *Bacteroides*. Both are bile-tolerant bacteria hence they are capable of digesting high-fat diets such as sow milk, which has a higher fat content (7.5–10.1%) compared with cow’s milk (4.2%) or human milk (3.2–3.6%)^29–32^. *Alistipes* had been reported to produce sulfonolipids, which play an important role in cell signaling and morphogenesis in animals, in mice fed with high-fat diets^33^. *Bacteroides* in infants were described to utilize human milk oligosaccharides (HMOs), bioactive components of milk that could not be completely digested by the host, using mucus-utilization pathways^34^. HMOs played several important roles in neonatal development, such as being prebiotics for gut microbiota and protecting the mucosal surface from pathogen binding, etc^35^. Similar to human milk, higher fucosylated compounds in porcine milk oligosaccharide were reported to be correlated with lower sialylated compounds at D.7 and D.14 of lactation compared with D.0 and a pre-colostrum period. The fucose consuming taxa containing genes encoding fucose-permease enzymes were also changed from *Enterobacteriaceae* in nursing pigs to *Lactobacillaceae* in weaned pigs^36^. Piglets are considered a good model for the study of human infant nutrition issues because of similar nutritional physiology such as a postnatal development of intestinal microbiota, the effect of gut microbiota diversity in early life on immune systems, and milk oligosaccharides^36,37^. In addition, *Bacteroides* in the piglet fecal microbiome has been shown to contain large amounts of genes encoding key catalytic enzymes such as sialidases and beta-hexosaminidases, that could digest sow milk glycans^38^. Clostridium was the major genus at D.0 and D.3, whilst the predominantly polysaccharide-degrading *Prevotella* was the major population at D.8. Soluble-fiber-degrading *Treponema* was also observed at higher abundance at D.8. Our results were similar to a previous report of *Prevotella* being markedly increased in weaned animals, supplanting the *Bacteroides* population^38^. The dynamic of the fecal microbiome population has been reported in antibiotic-free nursing and 7-day post-weaning piglets. *Bacteroides* was reported to be the most abundant microbe in nursing pigs, whereas in weaned piglets, *Prevotella* was enriched^39^. In our study, not only weaning age but diet was also shown to be associated with the microbial community composition. The same pre-starter (nursery) feed was provided throughout this study at the age of day 7–day 32 (D.8) (Table 5). After D.8, pigs would receive another feed formula of solid starter feed. Hence our diet factor that affected microbiota community was possibly a consequence from sow milk exclusion on the weaning day and more consumption of pre-starter feed. In the previous report of piglets receiving creep feed on D.14 and being weaned on D.28, age and diet were shown to play an important role in the bacterial community, with the most diversity at 21 days post weaning^40^. In general, during the weaning period, ETEC often flare up and cause diarrhea and other symptoms due to impaired immune functions. However, for the farms from which we collected fecal samples, diarrhea was rarely observed and *E. coli* was found to be lower at D.8, indicating good farm management, which was probably due to chlorination of drinking water for disinfection and the evaporative cooling housing system. Chlorination was shown to have a bactericidal effect on *E. coli*^41^. And in the housing equipped with evaporative cooling system, temperature and humidity of the housing are well controlled, leading to reduced heat stress and good health^42^. *Lactobacillus* was enriched at D. − 3 (1.17%). However, low amounts were observed at all ages (0.22–1.17%) despite being fed probiotics, containing *Lactobacillus acidophilus* and *Lactobacillus plantarum*, at postnatal D.1–3. According to a previous report, lactobacilli with probiotic potential were sensitive to several antibiotics used on the farms^43^. An antibiotic susceptibility test should be performed. On the other hand, low amounts of, or rare, probiotic microorganisms observed indicated that they did not serve as a reservoir for AMR determinants.

**Table 5:** Programs of feeds, antibiotics and probiotics for pregnant sows and piglets.

Feeds, antibiotics and probiotics	Day (Day 0 = Parturition day)
**1. Sows**	
400 ppm Chlortetracycline (dietary supplementation)	− 7 to − 6
100 ppm Tylvalosin (dietary supplementation)	− 7 to 7
10 mg/kg Kanamycin (intramuscular injection)	1–3
2. Piglets*	
Probiotics (dietary supplementation)**	1–3
15 mg/kg Amoxicillin (intramuscular injection)	7
Pre-starter feed	7–32
2.5 mg/kg Turathromycin (intramuscular injection)	21–23
400 ppm Amoxicillin and 250 ppm Neomycin (dietary supplementation)	25–32

*Ad libitum chlorine in water (1 tablet/ 1000 L water) was allowed.

**Probiotics (Bactosac, K.M.P. Biotech, Chon Buri, Thailand): 10^9^ colony-forming unit (CFU) each of *B. subtilis*, *B. licheniformis*, *L. acidophilus*, *L. plantarum*, *Pediococcus pentosaceus* and *Sacchalomyces cerevisiae*.

For the AMR patterns, the highest abundance of AMR determinants at D. − 3–D.3 conferred resistance to AMG, including the streptothricin acetyltransferase gene (*sat*) and *aac*(6')*-aph*(2''), encoding a bifunctional enzyme, AAC(6′)-APH(2"). *Sat* and *aac*(6')*-aph*(2'') encoded enzymes linked to the antibiotic inactivation mechanism by acetyltransferase. *sat* conferred resistance to streptothricin via acetylation of the drug^44^. Streptothricin was not used on our farms but the other AMG, kanamycin, was intramuscularly injected into the sows on parturition day, and neomycin was mixed with feed for piglets at D.25–D.8. SAT has been shown to acetylate kanamycin and neomycin at lower levels than streptothricin^45^. From our study, high abundance of *sat* was observed at D.8, although lower than at D. − 3–D.3 (Supplementary Table S2). For *aac*(6′)*-aph*(2″), it was carried on transposons, causing AMG resistance not only in Gram-positive bacteria, but also in Gram-negative *E. coli* clinical isolates^46^. From the CCA plot in our study, we found correlation of *aac*(6')*-aph*(2'') with *E. coli* in the D. − 3–D.3 group (Fig. 3B).

The other group with increased AMR determinants at D. − 3, D.0 and/or D.3, although at a lesser abundance than the AMG, conferred an MDR pattern such as *gad*W, *mdt*E*, emr*A, *evg*S*, tol*C and *mdt*B (Supplementary Table S2). *emr*A encodes the EmrA periplasmic adaptor of a multidrug efflux pump belonging to the major facilitator superfamily (MFS) efflux pumps. The EmrA periplasmic adaptor has been reported to link the outer membrane protein TolC as an EmrAB–TolC tripartite efflux system in *E. coli*, similar to that of AcrAB–TolC complex, which is in the resistance-nodulation-cell division (RND) family of transporters^47^. EmrAB–TolC and AcrAB–TolC complexes have been reported to play an important role in the tolerance of *E. coli* to bile salts in the GI tract^48^. In our study, we observed high abundance of *tol*C and *acr*B. We also observed higher abundance of *mdt*B at D. − 3–D.3. *mdt*B encodes MdtB, a part of the MdtABC system, which is in the RND family similar to the AcrAB–TolC system^49^. We also found high abundance of *mdt*E. MdtE is part of the MdtEF–TolC tripartite efflux system, conferring resistance to several compounds such as erythromycin, cloxacillin and oxacillin^50^. We also found high *cat*B abundance at D. − 3–D.3 (Fig. 3B, Supplementary Table S2). Plasmid-encoded *cat*B and *cml*A are known to cause chloramphenicol resistance^51^. Chloramphenicol has been prohibited for use in food-producing animals (http://www.farad.org/prohibited-and-restricted-drugs.html; accessed 10 November 2020). Both genes have been previously reported in *E. coli*, corresponding to our CCA plot results (Fig. 3D)^51^. Since this antibiotic and its derivatives have not been used in nursing and growing/finishing farms, the possibility of *E. coli* to be a reservoir for these AMR genes should be investigated further.

On the other hand, the highest abundance of AMR determinant at D.8 was *mef*A in the MLS resistance pattern (Supplementary Table S2). *mef*A is in the MFS efflux pump superfamily. In fact, *mef*A confers resistance to macrolide and has been reported to locate in transposons. Hence, it can be possibly transferred between bacterial species^52^. The high levels of *mef*A at all ages, particularly at D.8, might lead to a concern of macrolide usage on the farm, tylvalosin for sows at D. − 7–D.7 and turathromycin for piglets at D. − 3–D.23. However, the AMR genotype might not reflect its phenotypic resistance as the *erm*F gene, another AMR gene in the MLS resistance pattern, detected in various human clinical specimens, did not show clindamycin- or erythromycin-resistant phenotypes^53^. Hence, the macrolide resistance phenotype needs to be investigated further. *rlm*N encodes the methyltransferase RlmN, which is a member of the radical SAM enzyme superfamily and is involved in the methylation of 23S rRNA. The effect of *rlmN* mutation on the AMR is still obscure^54^. At D.8, high abundance of AMR determinants conferred resistance to β-lactam (*cfx*A6 and *aci*1) was also observed. The *cfx*A6 gene was positively correlated with *Prevotella* (Fig. 3F, Supplementary Table S3). *cfx*A was the most abundant β-lactam gene family in pigs, followed by *aci*^55^. *cfx*A has been reported in most β-lactamase-positive *Prevotella* strains^56^. However, another report revealed that approximately half of all *Prevotella* strains containing *cfx*A genes were resistant to β-lactam antibiotics^57^. In our case, as *Prevotella* was the most predominant bacterial population at D.8 owing to the post-weaning farm management practice (i.e. feed changes to solid feed), the associated AMR gene abundance was high as well. The occurrence of *cfx*A in β-lactamase-positive and negative *Prevotella* strains should be investigated further.

Tetracyclines are bacteriostatic antibiotics with broad spectrum activity. Tetracycline has been shown to be a selective pressure in fattening pigs, resulting in a greater number of tetracycline-resistant isolates^23^. In this study, *tet*(40) presents at the highest relative abundances across all samples, following by *tet*W. It encodes tetracycline efflux transporters under a MFS antibiotic efflux pump AMR gene family^58^. *tet*(40) is commonly found in pig gut microbiome. *tet*(40) from feces of organic pigs could present as a single tetracycline-resistance gene on a putative transposon and could be linked to *tet*W on a putative plasmid^58^. High *tet*(40) abundance has been reported in healthy sows not receiving antibiotics since weaning, probably reflecting the natural resistome^59^. In our study, sows were treated with chlortetracycline in feed at D. − 7–D. − 6 before parturition, whereas piglets from birth did not receive any tetracycline drug or its derivatives. However, residues of tetracycline and its derivatives have been reported in human breast milk^60^. The detection of antimicrobial residues in sow milk and of reservoirs of AMR genes in environments, such as soil and water, and the transmission of AMR genes among the microbiome should be investigated further. A limitation of the present study is the lack of phenotypic–genotypic comparison of resistance which is not available for frozen fecal samples.

The maintenance of AMR determinants on the farm is a complex problem. Several factors are involved, such as selective pressure, co-selection whereby the use of one antibiotic causes resistance to other drugs, and horizontal and vertical transmission of AMR determinants in the chromosome, plasmid, transposon or integron by bacteria^61^. As several antibiotics have been used on the farm for decades, such as tetracycline and AMG, and a number of strong positive correlations were observed, co-selection should be a concern. However, the withdrawal of antibiotics might not be able to solve such problems, as AMR determinants still presented even though pigs did not receive antibiotic, probably due to mobile genetic elements in the ecosystem^58^.

In order to resolve the AMR concerns in the weaning pigs, manipulation of gut microbiota has been suggested. Due to the absence of maternal immunity in weaning piglets, biotic supplements including probiotics, fermented prebiotics and synbiotics should be considered to strengthen gut microbiota. In neonatal piglets and pregnant and lactating sows fed with prebiotics-supplemented diets, the activity of bacteria and intestinal immunity, in addition to immunity in piglets were significantly increased^62^. The strategy of using these biotics was to replace the AMR microorganism communities with good microorganism populations. Another technique that has been successful in treating diseases relating to gut microbiota dysbiosis in humans is fecal microbiota transplantation (FMT). FMT has been reported to decolonize several AMR pathogens such as methicillin-resistant bacteria, extended spectrum β-lactamase (ESBL)-producing Enterobacteriaceae and vancomycin-resistant enterococci^63^.

## Conclusions

This study unveiled alteration of microbiome and AMR determinants in piglets at D. − 3–D.8 and that at D.8 or 8 days after weaning, piglets showed a different microbiome community and AMR determinants compared with other ages, reflecting the effects of weaning activity, dietary changes to a solid diet and farm transferring. The tracking of AMR gene abundance in other pig ages such as fattening pigs, slaughterhouse, pork in the markets and pig farmers should be investigated.

## Materials and methods

### Statement

All methods were carried out in accordance with guidelines and regulations and the study was carried out in compliance with the ARRIVE guidelines. All animals were managed following the ethical guidelines required under the Chulalongkorn University Animal Care and Use Committee (CU-ACUC), Thailand (approval number 1731036).

### Animals and sampling

A total of twenty-four crossbred Landrace × Large White × Duroc Jersey piglets from three sows raised on a local commercial farm were used in the study. Pigs were raised under the evaporative cooling housing system which can control air temperature and humidity in a building. The programs of feeds, antibiotics and probiotics for pregnant sows and piglets are shown in Table 5. All antimicrobial prophylaxis given to sows or piglets was at therapeutic doses and was used for a short-term period only (≤ 14 days), hence, they did not serve as growth promoters. Weaning pigs were transferred to an early weaning unit in a weaner/finisher farm. Fecal samples were collected from the rectum of each pig at D. − 3 (before receiving tulathromycin), D.0, D.3 and D.8, delivered to the laboratory under dry ice and stored at − 80 °C until DNA extraction was performed.

### DNA extraction

DNA was extracted from 180 mg fecal samples using a QIAamp Fast DNA Stool Mini kit (Qiagen, Hilden, Germany) according to the manufacturer’s protocol. Genomic DNA was cleaned and concentrated by a Genomic DNA Clean & Concentrator Kit-10 (Zymo Research, Irvine, CA, USA), according to the manufacturer’s directions. DNA concentration and purity were determined using a NanoDrop ND-1000 Spectrophotometer V3.7 (Thermo Fisher Scientific, Waltham, MA, USA) and the integrity of total DNA samples was evaluated with a 2100 Bioanalyzer (Agilent Technologies, Santa Clara, CA, USA). DNA samples were stored at − 20 °C until further analysis.

### DNA library preparation and metagenomic sequencing

DNA library preparation was performed according to the TruSeq Nano DNA Sample Preparation Kit protocol (Illumina, San Diego, CA, USA). The samples were sheared on a Covaris S220 or E220 focused ultrasonicator (Covaris, Woburn, MA, USA) to approximately 450 bp, following the manufacturer’s recommendation, and uniquely tagged with one of the TruSeq LT DNA barcodes (Illumina). The finished libraries were quantitated using a Quant-iT PicoGreen dsDNA Kit (Thermo Fisher Scientific) and the average library size was determined using a Bioanalyzer 2100 instrument, using a DNA 7500 chip (Agilent Technologies). Library concentrations were then normalized to 4 nM and validated by qPCR on a ViiA-7 real-time thermocycler (Thermo Fisher Scientific), using the KAPA Library Quantification Kit for Illumina platforms (Roche, Basel, Switzerland). The libraries were then pooled at equimolar concentrations and sequenced on an Illumina HiSeq2500 sequencer in rapid mode at a read length of 250 bp (paired-end read).

### Data analysis

The sequencing reads for all samples were trimmed using Trimmomatic version 0.38^64^. DIAMOND version 0.9.10.111 was used to map the trimmed read pairs to the NCBI non-redundant (NR) protein database, retrieved on June 8, 2018^65^. An operational taxonomic unit (OTU) table was constructed from the taxonomic classification results with a 97% confidence threshold. OTUs with their total number of mapped reads across all samples occupying less than 0.005% of overall mapped reads were filtered out, which was then subsampled to 1 262 472 reads per sample. This subsampling value is 80% of the sample with the lowest number of paired reads after trimming. The OTU table filtering and subsampling, in addition to subsequent alpha and beta diversity analyses, were performed using the QIIME 2 platform (https://qiime2.org/)^66^. A principal coordinates analysis (PCoA) plot of fecal microbiome taxonomy was constructed based on a pairwise dissimilarity matrix.

The reference AMR gene sequences were obtained by integrating the ResFinder version 3.2 (https://cge.cbs.dtu.dk/services/ResFinder/; accessed 21 September 2018)^67^, ARG-ANNOT version 4^68^, and CARD version 2.0.3 databases using ARGDIT^69,70^. This reference sequence set was then clustered using CD-HIT with a 90% sequence identity threshold^71^. The trimmed read pairs were mapped to the reference AMR gene sequences using Bowtie 2^72^ and those mapped pairs were assigned to the reference AMR gene sequence clusters accordingly. The gene abundances in fragments per kilobase of exon model per million mapped reads (FPKM) were then calculated from the mapped read counts of the clusters. Any cluster with its total abundance across all samples lower than 0.005% of the overall abundance was discarded. A stacked bar graph showing relative abundance distribution of antimicrobial resistance genes by antimicrobial drug resistance patterns was constructed from the proportion of read counts from the ResFinder annotation and presented using Microsoft Excel. Beta diversity analysis and a PCoA plot were performed for AMR abundance as for taxonomic abundance.

Canonical correlation analysis (CCA) plots showed interrelationships between taxon profile and AMR profiles, using the regularized version implemented in the R package CCA because of there being a larger number of variables (taxa or AMR sequences) than samples^73^. The association of AMR genes, bacterial community structure at the genus level and pig samples in each antimicrobial drug resistance pattern was analyzed using the vegan R package.

### Statistical analyses

For DIAMOND results of microbiome communities, two statistical analyses were performed between groups of samples, namely: (1) permutational multivariate analysis of variance (PERMANOVA) to examine differences in microbiome community composition among groups of samples, under the null hypothesis of no differences among groups. PERMANOVA was performed using the adonis function in the R package vegan (version 2.5-2)^74^; (2) Alpha diversity of the samples was estimated using the Shannon index. Compositional difference between any two samples (beta diversity) was quantified with Bray–Curtis dissimilarity, and the PCoA plot at the family level was computed from the pairwise dissimilarity matrix obtained. This PCoA plot was visualized through the Emperor plugin for QIIME 2^75^. Both PERMANOVA and alpha diversity calculations employed uniformly subsampled data, as described above.

For both taxa and detected AMR determinants, we tested for differences in relative abundance of each taxon or AMR sequence entity, respectively, among groups using generalized linear models (GLM) implemented in the R package mvabund^76^. Statistical analyses of Shannon diversity index and relative abundance of microbial phyla and microbial genera were performed using the Kruskal–Wallis test. For AMR gene data, we employed the function manyglm with a negative binomial distribution and with the offset component set to the log of the sum of FPKM values for each sample. Day of sampling was treated as the factor of interest. We estimated *P*-values using the anova.manyglm function, using 1000 bootstrap resamples (resample parameters set to ‘PIT-trap’) and we unadjusted for multiple hypothesis testing using the step-down procedure specified by the parameter uni.p set to "adjusted". All other parameters in anova.manyglm had default settings. Differential abundance of AMR genes corresponding to antimicrobial drug resistance patterns was analyzed using the DESeq2 version 1.30.0 in R software^77^, and data were visualized using ggplot2 version 3.2.1 in the tidyverse package (https://CRAN.R-project.org/package=tidyverse/; accessed 10 October 2020).

### Ethics approval and consent to participate

The study was approved by the Chulalongkorn University Animal Care and Use Committee (CU-ACUC), Bangkok, Thailand (approval number 1731036).

## Supplementary Information

Supplementary Information.

## Data Availability

Additional data is available in the supplementary material, and the datasets generated during the current study are available in the Sequence Read Archive repository, in the BioProject PRJNA662672 (https://www.ncbi.nlm.nih.gov/sra/PRJNA 66267 2).
