# Supplementary material for: Dynamics of the fecal microbiome and antimicrobial resistome in commercial piglets during the weaning period
Source: Sci Rep. 2021 Sep 10;11:18091. doi: 10.1038/s41598-021-97586-9 (PMC8433359; doi:10.1038/s41598-021-97586-9)

**Table S1.** The relative abundance of genera (≥0.1% in at least one group) in piglets at day 3 pre-weaning (D.−3), weaning day (D.0), day 3 post-weaning (D.3) and day 8 post-weaning (D.8)

| Taxonomy | Relative frequency (%) ± SD | | | | | | | |
| --- | --- | --- | --- | --- | --- | --- | --- | --- |
|  | D.−3 | | D.0 | | D.3 | | D.8 | |
| *Alistipes* | 12.69 | ± 9.99^a^ | 6.86 | ± 4.90^ab^ | 7.04 | ± 7.30^b^ | 6.01 | ± 6.13^b^ |
| *Bacteroides* | 8.54 | ± 6.66^a^ | 8.23 | ± 5.68^a^ | 5.22 | ± 2.97^a^ | 2.73 | ± 0.79^b^ |
| *Prevotella* | 7.18 | ± 7.32^a^ | 6.96 | ± 6.31^a^ | 3.63 | ± 2.00^a^ | 20.88 | ± 11.28^b^ |
| *Clostridium* | 4.68 | ± 4.51^ab^ | 7.98 | ± 5.38^a^ | 6.84 | ± 5.05^a^ | 2.84 | ± 2.21^b^ |
| *Parabacteroides* | 1.20 | ± 0.70^a^ | 1.20 | ± 0.80^a^ | 2.73 | ± 3.08^b^ | 0.37 | ± 0.55^c^ |
| *Lactobacillus* | 1.17 | ± 1.69^a^ | 0.22 | ± 0.18^b^ | 0.34 | ± 0.44^b^ | 0.96 | ± 2.04^ab^ |
| *Eubacterium* | 0.84 | ± 0.91^a^ | 0.48 | ± 0.44^ab^ | 0.34 | ± 0.30^b^ | 0.63 | ± 0.33^a^ |
| *Pyramidobacter* | 0.83 | ± 2.48^a^ | 0.25 | ± 0.73^a^ | 0.13 | ± 0.13^a^ | 0.02 | ± 0.05^b^ |
| *Butyricimonas* | 0.81 | ± 0.65^ab^ | 1.17 | ± 1.40^a^ | 0.43 | ± 0.33^b^ | 0.10 | ± 0.08^c^ |
| *Phascolarctobacterium* | 0.57 | ± 0.42^a^ | 0.73 | ± 1.01^a^ | 0.26 | ± 0.23^b^ | 0.61 | ± 0.27^a^ |
| *Treponema* | 0.49 | ± 0.78^a^ | 1.67 | ± 2.42^b^ | 1.16 | ± 1.32^ab^ | 2.64 | ± 1.95^c^ |
| *Fusobacterium* | 0.48 | ± 1.07^ab^ | 0.49 | ± 1.09^a^ | 0.04 | ± 0.05^a^ | 0.02 | ± 0.04^b^ |
| *Oscillibacter* | 0.47 | ± 0.33^a^ | 0.66 | ± 0.39^ab^ | 0.54 | ± 0.20^a^ | 0.82 | ± 0.33^b^ |
| *Escherichia* | 0.41 | ± 0.61^a^ | 0.23 | ± 0.27^a^ | 0.34 | ± 0.49^a^ | 0.06 | ± 0.15^b^ |
| *Desulfovibrio* | 0.37 | ± 0.24^a^ | 0.33 | ± 0.17^a^ | 0.55 | ± 0.34^a^ | 0.10 | ± 0.08^b^ |
| *Anaeromassilibacillus* | 0.34 | ± 0.38^a^ | 0.41 | ± 0.67^a^ | 0.80 | ± 0.70^b^ | 0.05 | ± 0.07^c^ |
| *Roseburia* | 0.28 | ± 0.80^a^ | 0.14 | ± 0.13^ab^ | 0.11 | ± 0.10^a^ | 0.46 | ± 0.58^b^ |
| *Chlamydia* | 0.25 | ± 0.23^a^ | 0.40 | ± 0.49^a^ | 0.34 | ± 0.43^a^ | 0.58 | ± 0.33^b^ |
| *Lachnoclostridium* | 0.24 | ± 0.18^a^ | 0.23 | ± 0.26^a^ | 0.23 | ± 0.16^a^ | 0.09 | ± 0.04^b^ |
| *Faecalibacterium* | 0.23 | ± 0.28^a^ | 0.26 | ± 0.18^ab^ | 0.43 | ± 0.48^b^ | 0.61 | ± 0.36^c^ |
| *Anaerotruncus* | 0.22 | ± 0.17^a^ | 0.11 | ± 0.09^b^ | 0.12 | ± 0.16^b^ | 0.06 | ± 0.04^c^ |
| *Subdoligranulum* | 0.20 | ± 0.35^a^ | 0.14 | ± 0.16^a^ | 0.38 | ± 0.51^b^ | 0.05 | ± 0.03^c^ |
| *Flavonifractor* | 0.19 | ± 0.15^a^ | 0.23 | ± 0.20^ab^ | 0.23 | ± 0.09^b^ | 0.15 | ± 0.07^a^ |
| *Odoribacter* | 0.16 | ± 0.21^a^ | 0.08 | ± 0.06^a^ | 0.09 | ± 0.05^a^ | 0.03 | ± 0.06^b^ |
| *Pseudoflavonifractor* | 0.11 | ± 0.08^ab^ | 0.12 | ± 0.08^a^ | 0.13 | ± 0.05^a^ | 0.08 | ± 0.05^b^ |
| *Culturomica* | 0.11 | ± 0.26^ab^ | 0.02 | ± 0.06^a^ | 0.04 | ± 0.05^b^ | 0.02 | ± 0.08^c^ |
| *Veillonella* | 0.10 | ± 0.11^a^ | 0.10 | ± 0.12^ab^ | 0.07 | ± 0.10^b^ | 0.02 | ± 0.04^c^ |
| *Cloacibacillus* | 0.09 | ± 0.11^ab^ | 0.08 | ± 0.11^a^ | 0.34 | ± 0.51^b^ | 0.03 | ± 0.05^c^ |
| *Intestinimonas* | 0.09 | ± 0.06^a^ | 0.08 | ± 0.05^a^ | 0.11 | ± 0.04^b^ | 0.06 | ± 0.04^a^ |
| *Blautia* | 0.07 | ± 0.04^a^ | 0.08 | ± 0.03^a^ | 0.13 | ± 0.05^b^ | 0.16 | ± 0.17^b^ |
| *Sutterella* | 0.07 | ± 0.07^a^ | 0.23 | ± 0.32^b^ | 0.04 | ± 0.04^a^ | 0.06 | ± 0.05^a^ |
| *Fibrobacter* | 0.05 | ± 0.17^a^ | 0.04 | ± 0.05^ab^ | 0.06 | ± 0.07^b^ | 0.22 | ± 0.23^c^ |
| *Butyricicoccus* | 0.04 | ± 0.02^a^ | 0.04 | ± 0.02^ab^ | 0.08 | ± 0.09^bc^ | 0.10 | ± 0.11^c^ |
| *Sphaerochaeta* | 0.04 | ± 0.07^a^ | 0.13 | ± 0.22^b^ | 0.10 | ± 0.16^b^ | 0.12 | ± 0.09^b^ |
| *Streptococcus* | 0.04 | ± 0.02^a^ | 0.05 | ± 0.04^a^ | 0.16 | ± 0.29^b^ | 0.05 | ± 0.04^a^ |

^a, b, c^ denote a significant difference in the same row at *P* < 0.05; Kruskall-Wallis test

**Table S2.** The relative abundance of antimicrobial resistance genes (≥0.1% in at least one group) in piglets at day 3 pre-weaning (D.−3), weaning day (D.0), day 3 post-weaning (D.3) and day 8 post-weaning (D.8)

| Antimicrobial resistance genes |  | Relative frequency (%) ± SD | | | | | | | |
| --- | --- | --- | --- | --- | --- | --- | --- | --- | --- |
|  |  | D.−3 | | D.0 | | D.3 | | D.8 | |
| *sat* | Aminoglycosides | 1822.44 | ± 38.87^e^ | 2012.06 | ± 33.26^e^ | 2196.3 | ± 35.76^e^ | 685.15 | ± 13.83^f^ |
| *aac*(6')*-aph*(2'') | Aminoglycosides | 1416.92 | ± 32.22^e^ | 1131.01 | ± 23.91^e^ | 1545.89 | ± 29.26^e^ | 296.23 | ± 7.70^f^ |
| *spw* | Aminoglycosides | 1036.95 | ± 24.37 | 807.38 | ± 15.58^e^ | 647 | ± 13.39^e^ | 1217.88 | ± 30.74^f^ |
| bifunctional_aminoglycoside_modifying_enzyme | Aminoglycosides | 940.7 | ± 18.93^e^ | 779.81 | ± 12.09 | 1171.93 | ± 22.48^e^ | 308.59 | ± 6.60^f^ |
| *aad*S | Aminoglycosides | 683.96 | ± 24.54^e^ | 388.38 | ± 10.98 | 438.5 | ± 17.91 | 116.51 | ± 3.65^f^ |
| *aad*A | Aminoglycosides | 193.76 | ± 8.91^c,e^ | 114.41 | ± 4.46^e^ | 75.78 | ± 3.03^d^ | 17.14 | ± 0.70^f^ |
| *str*A | Aminoglycosides | 140.85 | ± 6.62^e^ | 81.52 | ± 4.34 | 99.63 | ± 4.04 | 14.35 | ± 1.17^f^ |
| *str*B | Aminoglycosides | 137.8 | ± 6.56^e^ | 77.6 | ± 3.19 | 85.64 | ± 3.55 | 11.26 | ± 1.21^f^ |
| *acr*F | Aminoglycosides | 68.28 | ± 4.21^e^ | 40.26 | ± 2.03^e^ | 39.14 | ± 2.38^c^ | 2.04 | ± 0.30^d,f^ |
| *aac*(3)-VI | Aminoglycosides | 31.95 | ± 2.03^c^ | 12.94 | ± 0.79 | 15.31 | ± 1.29 | 0 | ± 0.00^d^ |
| *sph* | Aminoglycosides | 3.1 | ± 0.23^a^ | 28.17 | ± 2.96^b^ | 9.05 | ± 0.78 | 4.69 | ± 0.36 |
| *cfx*A5 | Beta-lactams | 630.44 | ± 16.84 | 873.41 | ± 30.07 | 468.3 | ± 8.15^e^ | 787.37 | ± 15.59^f^ |
| *ORF*3 | Beta-lactams | 471.89 | ± 26.57^e^ | 220.89 | ± 10.25^e^ | 136.03 | ± 12.83 | 26.79 | ± 2.70^f^ |
| *fus*1 | Beta-lactams | 164.6 | ± 19.63^a^ | 122.57 | ± 12.58^c^ | 8.54 | ± 0.54 | 0.35 | ± 0.07^b,d^ |
| *cfx*A6_beta | Beta-lactams | 120.62 | ± 10.28^e^ | 161.04 | ± 6.39^e^ | 61.63 | ± 2.88^e^ | 700.99 | ± 23.08^f^ |
| *aci*1 | Beta-lactams | 64.76 | ± 2.28^e^ | 133.56 | ± 5.16 | 85.38 | ± 3.00^e^ | 252.75 | ± 8.20^f^ |
| *cep*A | Beta-lactams | 59.4 | ± 4.43^e^ | 39.71 | ± 2.14^e^ | 25.1 | ± 1.82 | 0.63 | ± 0.13^f^ |
| *mrd*A | Beta-lactams | 58.51 | ± 3.49^e^ | 35.47 | ± 2.38^e^ | 33.71 | ± 2.07^e^ | 1.81 | ± 0.28^f^ |
| *mef*A | MLS* | 1470.18 | ± 31.07^e^ | 1154.98 | ± 24.63^e^ | 943.70 | ± 14.73^e^ | 2361.62 | ± 49.22^f^ |
| *mef*A | MLS | 519.77 | ± 12.09^e^ | 446.26 | ± 9.17^e^ | 345.98 | ± 5.96^e^ | 884.59 | ± 17.90^f^ |
| *mph*B | MLS | 14.50 | ± 0.85 | 21.46 | ± 0.84 | 13.16 | ± 0.59^e^ | 32.86 | ± 1.23^f^ |
| *mef*B | MLS | 31.80 | ± 2.05^e^ | 32.94 | ± 1.65^e^ | 15.06 | ± 1.62 | 0.93 | ± 0.15^f^ |
| *nim*J | Metronidazole | 160.82 | ± 12.22 | 39.59 | ± 3.17 | 16.33 | ± 0.85^e^ | 59.27 | ± 2.47^f^ |
| *qac*H | Multidrug | 104.34 | ± 5.81^e^ | 71.9 | ± 4.58 | 19.65 | ± 2.02 | 2.61 | ± 0.39^f^ |
| transcriptional_  regulator | Multidrug | 96.72 | ± 4.83^e^ | 56.17 | ± 3.10^e^ | 50.46 | ± 3.09 | 3.11 | ± 0.50^f^ |
| *gad*W | Multidrug | 87.98 | ± 5.75^e^ | 52.24 | ± 2.92^e^ | 52.73 | ± 3.14^e^ | 1.94 | ± 0.33^f^ |
| *mdt*E | Multidrug | 84.32 | ± 5.17^e^ | 47.85 | ± 2.68^e^ | 45.6 | ± 2.75^e^ | 1.5 | ± 0.31^f^ |
| *emr*A | Multidrug | 73.92 | ± 4.19^e^ | 38.46 | ± 2.06^e^ | 40.5 | ± 2.59^e^ | 1.48 | ± 0.30^f^ |
| *evg*S | Multidrug | 72.11 | ± 4.45^e^ | 38.71 | ± 2.15^e^ | 40.54 | ± 2.53^e^ | 1.67 | ± 0.23^f^ |
| *tol*C | Multidrug | 69.08 | ± 4.16^e^ | 39.53 | ± 2.09^e^ | 40.3 | ± 2.61^e^ | 1.94 | ± 0.36^f^ |
| *acr*B | Multidrug | 60.19 | ± 3.50^e^ | 34.68 | ± 1.96^e^ | 33.39 | ± 2.05 | 2.11 | ± 0.30^f^ |
| *mdt*B | Multidrug | 56.37 | ± 3.11^e^ | 37.72 | ± 1.96^e^ | 39.09 | ± 2.32^e^ | 2.38 | ± 0.28^f^ |
| *rlm*N | Phenicol | 1113.56 | ± 30.40^e^ | 1146.12 | ± 24.17^e^ | 1319.75 | ± 23.82^e^ | 1380.01 | ± 19.75^f^ |
| *cat*B4 | Phenicol | 357.86 | ± 20.17^e^ | 187.52 | ± 10.54^e^ | 181.00 | ± 9.39^e^ | 9.95 | ± 1.71^f^ |
| *cml*A4 | Phenicol | 65.02 | ± 4.33^e^ | 46.86 | ± 2.26^e^ | 14.64 | ± 1.13 | 0.73 | ± 0.11^f^ |
| *PP* | Phenicol | 56.14 | ± 4.44^e^ | 25.90 | ± 1.52^e^ | 17.69 | ± 1.74 | 0.00 | ± 0.00^f^ |
| *sul*3 | Sulfonamides | 123.12 | ± 8.03^e,g^ | 68.37 | ± 3.25^g^ | 26.04 | ± 1.60^f^ | 5.38 | ± 0.31^h^ |
| *sul*1 | Sulfonamides | 19.68 | ± 1.09^e^ | 20.12 | ± 1.34^e^ | 10.07 | ± 0.49 | 0.97 | ± 0.11^f^ |
| *tet*(40) | Tetracyclines | 600.97 | ± 17.06^e^ | 785.62 | ± 18.22 | 1338.81 | ± 24.19^f^ | 761.35 | ± 13.18^f^ |
| *tet*W | Tetracyclines | 149.83 | ± 4.40 | 238.40 | ± 5.79^e^ | 200.76 | ± 4.36 | 73.04 | ± 1.49^f^ |
| *tet*T | Tetracyclines | 28.76 | ± 1.44^e^ | 7.62 | ± 0.80 | 2.90 | ± 0.53 | 0.00 | ± 0.00^f^ |
| *tet*A(B) | Tetracyclines | 17.99 | ± 0.99^e^ | 18.35 | ± 1.22^e^ | 4.34 | ± 0.39 | 0.24 | ± 0.05^f^ |
| *dhfr*XII | Trimethoprim | 77.56 | ± 5.64^e^ | 28.68 | ± 1.81 | 17.47 | ± 1.80 | 0.00 | ± 0.00^f^ |

* MLS: Macrolide-Lincosamide-Streptogramin

^a-b^ denote a significant difference in the same row at *P* < 0.05; DESeq2 test

^c-d^ denote a significant difference in the same row at *P* < 0.01; DESeq2 test

^e-f^ and ^g-h^ denote a significant difference in the same row at *P* < 0.001; DESeq2 test

**Table S3** Statistical significance test of the CCA model using permutation test under reduced model. Pr(>F) is the *P*-value associated with the *F* statistic of a CCA model.

|  | **Degrees of *freedom* (Df)** | **Chi-Square** | **F-distributions** | **Pr(>F)** |  |
| --- | --- | --- | --- | --- | --- |
| **1. A CCA model of aminoglycoside resistance genes** | | | | |  |
| 1.1. Testing the significance of the CCA model | | | | |  |
| Model | 36 | 0.7451 | 2.998 | 0.001 |  |
| Residual | 59 | 0.4073 | NA | NA |  |
| 1.2. Testing the significance of terms (environmental variables) | | | | | |
| *aac*(6')*-aph*(2'') | 1 | 0.07431 | 10.7662 | 0.001 | *** |
| *aad* | 1 | 0.05435 | 7.8741 | 0.001 | *** |
| *aad*A5 | 1 | 0.03095 | 4.4837 | 0.001 | *** |
| *aad*E | 1 | 0.08498 | 12.3118 | 0.001 | *** |
| *aad*S | 1 | 0.03728 | 5.401 | 0.001 | *** |
| *ant*(6) | 1 | 0.0512 | 7.4182 | 0.001 | *** |
| *aac*(3) | 1 | 0.02702 | 3.9141 | 0.004 | ** |
| *aac*(6) | 1 | 0.0262 | 3.7959 | 0.002 | ** |
| *aad*K | 1 | 0.03832 | 5.5516 | 0.009 | ** |
| *acr*E | 1 | 0.02015 | 2.9185 | 0.006 | ** |
| *hph* | 1 | 0.02364 | 3.4251 | 0.003 | ** |
| *kdp*E | 1 | 0.02541 | 3.6808 | 0.002 | ** |
| *aac*A4 | 1 | 0.01914 | 2.7727 | 0.026 | * |
| *aad*E | 1 | 0.01765 | 2.5576 | 0.018 | * |
| *acr*D | 1 | 0.01401 | 2.0296 | 0.044 | * |
| Residual | 59 | 0.40725 |  |  |  |
| 1.3. Testing the significance of CCA axes | | | |  |  |
| CCA1 | 1 | 0.22472 | 33.6599 | 0.001 | *** |
| CCA2 | 1 | 0.15184 | 22.7434 | 0.001 | *** |
| CCA3 | 1 | 0.09573 | 14.3384 | 0.001 | *** |
| CCA4 | 1 | 0.07292 | 10.9218 | 0.006 | ** |
| **2. A CCA model of beta-lactam resistance genes** | | | |  |  |
| 2.1. Testing the significance of the CCA model | | | |  |  |
| Model | 21 | 0.5933 | 3.7397 | 0.001 |  |
| Residual | 74 | 0.5590 | NA | NA |  |
| 2.2. Testing the significance of terms (environmental variables) | | | |  |  |
| *aci*1 | 1 | 0.08121 | 10.7493 | 0.001 | *** |
| *amp*C | 1 | 0.05472 | 7.2427 | 0.001 | *** |
| *bla* | 1 | 0.04349 | 5.7566 | 0.001 | *** |
| *cfx*A5 | 1 | 0.06324 | 8.3703 | 0.001 | *** |
| *cfx*A6 | 1 | 0.09286 | 12.2913 | 0.001 | *** |
| *bla* | 1 | 0.03505 | 4.6398 | 0.002 | ** |
| *bla*CARB | 1 | 0.02637 | 3.4906 | 0.002 | ** |
| *fus*1 | 1 | 0.02648 | 3.5057 | 0.003 | ** |
| *omp*K37 | 1 | 0.02437 | 3.2264 | 0.004 | ** |
| *amp*H | 1 | 0.01533 | 2.0289 | 0.049 | * |
| *bla* | 1 | 0.03124 | 4.1354 | 0.011 | * |
| *cbl*A | 1 | 0.01941 | 2.5687 | 0.029 | * |
| Residual | 74 | 0.55905 |  |  |  |
| 2.3. Testing the significance of CCA axes | | | |  |  |
| CCA1 | 1 | 0.22615 | 29.9356 | 0.001 | *** |
| CCA2 | 1 | 0.14397 | 19.0564 | 0.001 | *** |
| CCA3 | 1 | 0.06726 | 8.9028 | 0.005 | ** |
| CCA4 | 1 | 0.05494 | 7.2719 | 0.018 | * |
| **3. A CCA model of macrolide-lincosamide-streptogramin (MLS) resistance genes** | | | | | |
| 3.1. Testing the significance of the CCA model | | | |  |  |
| Model | 29 | 0.6185 | 2.6370 | 0.001 |  |
| Residual | 66 | 0.5338 | NA | NA |  |
| 3.2. Testing the significance of terms (environmental variables) | | | | |  |
| *ABC* | 1 | 0.09135 | 11.2945 | 0.001 | *** |
| *erm*2 | 1 | 0.05427 | 6.7094 | 0.001 | *** |
| *erm*A | 1 | 0.0318 | 3.9317 | 0.001 | *** |
| *ere*D | 1 | 0.03106 | 3.8402 | 0.009 | ** |
| *erm* | 1 | 0.03033 | 3.7493 | 0.009 | ** |
| *erm*35 | 1 | 0.04122 | 5.096 | 0.002 | ** |
| *erm*47 | 1 | 0.03171 | 3.9208 | 0.002 | ** |
| *lin*G | 1 | 0.02652 | 3.2788 | 0.008 | ** |
| *lnu*C | 1 | 0.02326 | 2.8761 | 0.009 | ** |
| *mef*A | 1 | 0.02705 | 3.3439 | 0.003 | ** |
| *erm* | 1 | 0.01836 | 2.2702 | 0.033 | * |
| *erm*33 | 1 | 0.01895 | 2.3432 | 0.023 | * |
| *mef*A | 1 | 0.01672 | 2.0666 | 0.031 | * |
| *mph*B | 1 | 0.01931 | 2.3875 | 0.018 | * |
| *ole*B | 1 | 0.01872 | 2.3146 | 0.024 | * |
| Residual | 66 | 0.53382 |  |  |  |
| 3.3. Testing the significance of CCA axes | | | |  |  |
| CCA1 | 1 | 0.20581 | 25.4458 | 0.001 | *** |
| CCA2 | 1 | 0.12968 | 16.0332 | 0.001 | *** |
| CCA3 | 1 | 0.07668 | 9.4799 | 0.046 | * |
| **4. A CCA model of multidrug resistance genes** | | | |  |  |
| 4.1. Testing the significance of the CCA model | | | |  |  |
| Model | 46 | 0.7609 | 2.0709 | 0.001 |  |
| Residual | 49 | 0.3914 | NA | NA |  |
| 4.2. Testing the significance of terms (environmental variables) | | | |  |  |
| *acr*A | 1 | 0.07558 | 9.4618 | 0.001 | *** |
| *efr*A | 1 | 0.03831 | 4.7961 | 0.001 | *** |
| *mef*(En2) | 1 | 0.04192 | 5.2479 | 0.001 | *** |
| *acr*B | 1 | 0.02825 | 3.5368 | 0.003 | ** |
| *cpx*A | 1 | 0.03052 | 3.8213 | 0.002 | ** |
| *mdf*A | 1 | 0.02347 | 2.9381 | 0.007 | ** |
| *mdt*B | 1 | 0.0253 | 3.1669 | 0.003 | ** |
| *mdt*C | 1 | 0.02618 | 3.277 | 0.003 | ** |
| *msb*A | 1 | 0.02271 | 2.8431 | 0.01 | ** |
| *oqx*B | 1 | 0.02401 | 3.0055 | 0.005 | ** |
| *yoj*I | 1 | 0.02165 | 2.7102 | 0.007 | ** |
| *bae*S | 1 | 0.01963 | 2.4579 | 0.019 | * |
| *emr*A | 1 | 0.01707 | 2.1367 | 0.05 | * |
| *emr*Y | 1 | 0.01652 | 2.0685 | 0.039 | * |
| *mdt*H | 1 | 0.01719 | 2.152 | 0.038 | * |
| *mef*G | 1 | 0.01776 | 2.2228 | 0.025 | * |
| *mex*F | 1 | 0.0204 | 2.5533 | 0.011 | * |
| Residual | 49 | 0.39141 |  |  |  |
| 4.3. Testing the significance of CCA axes | | | |  |  |
| CCA1 | 1 | 0.20495 | 31.9406 | 0.001 | *** |
| CCA2 | 1 | 0.14601 | 22.7556 | 0.002 | ** |
| CCA3 | 1 | 0.10332 | 16.1029 | 0.042 | * |
| **5. A CCA model of phenicol resistance genes** | | | |  |  |
| 5.1. Testing the significance of the CCA model | | | |  |  |
| Model | 12 | 0.2663 | 2.0785 | 0.001 |  |
| Residual | 83 | 0.8861 | NA | NA |  |
| 5.2. Testing the significance of terms (environmental variables) | | | |  |  |
| *cml*A4 | 1 | 0.03892 | 3.6453 | 0.001 | *** |
| *cat*B4 | 1 | 0.05455 | 5.1102 | 0.002 | ** |
| *cml* | 1 | 0.03432 | 3.215 | 0.005 | ** |
| *rlm*N | 1 | 0.02677 | 2.5076 | 0.016 | * |
| 5.3. Testing the significance of CCA axes | | | |  |  |
| CCA1 | 1 | 0.11176 | 10.469 | 0.001 | *** |
| **6. A CCA model of sulfonamide resistance genes** | | | |  |  |
| 6.1. Testing the significance of the CCA model | | | |  |  |
| Model | 3 | 0.1372 | 4.1459 | 0.001 |  |
| Residual | 92 | 1.0151 | NA | NA |  |
| 6.2. Testing the significance of terms (environmental variables) | | | |  |  |
| *sul*3 | 1 | 0.06699 | 6.0717 | 0.001 | *** |
| *sul*2 | 1 | 0.043 | 3.8973 | 0.004 | ** |
| *sul*1 | 1 | 0.02724 | 2.4688 | 0.034 | * |
| Residual | 92 | 1.01511 |  |  |  |
| 6.3. Testing the significance of CCA axes | | | |  |  |
| CCA1 | 1 | 0.08127 | 7.3652 | 0.001 | *** |
| CCA2 | 1 | 0.03332 | 3.02 | 0.032 | * |
| **7. A CCA model of tetracycline resistance genes** | | | |  |  |
| 7.1. Testing the significance of the CCA model | | | |  |  |
| Model | 33 | 0.7297 | 3.2435 | 0.001 |  |
| Residual | 62 | 0.4227 | NA | NA |  |
| 7.2. Testing the significance of terms (environmental variables) | | | |  |  |
| *tet* | 1 | 0.0507 | 7.4366 | 0.001 | *** |
| *tet* | 1 | 0.04032 | 5.9139 | 0.001 | *** |
| *tet* | 1 | 0.04258 | 6.2454 | 0.001 | *** |
| *tet*(32) | 1 | 0.06888 | 10.1032 | 0.001 | *** |
| *tet*(40) | 1 | 0.057 | 8.3618 | 0.001 | *** |
| *tet*AA | 1 | 0.02743 | 4.0234 | 0.001 | *** |
| *tet*A(Q)2 | 1 | 0.05031 | 7.3796 | 0.001 | *** |
| *tet*M | 1 | 0.0328 | 4.8118 | 0.001 | *** |
| *tet*(W/N/W) | 1 | 0.02849 | 4.1786 | 0.001 | *** |
| *tet*(O/32/O) | 1 | 0.0204 | 2.9923 | 0.007 | ** |
| *tet*(44) | 1 | 0.0219 | 3.2132 | 0.005 | ** |
| *tet*OW | 1 | 0.02737 | 4.0147 | 0.003 | ** |
| *tet*T | 1 | 0.02551 | 3.7416 | 0.002 | ** |
| *tet*A | 1 | 0.01677 | 2.4603 | 0.031 | * |
| *tet*A | 1 | 0.01561 | 2.2893 | 0.03 | * |
| *tet*A(B) | 1 | 0.01647 | 2.4166 | 0.027 | * |
| *tet*G | 1 | 0.01649 | 2.4193 | 0.019 | * |
| *tet*M(916) | 1 | 0.01627 | 2.3868 | 0.019 | * |
| *tet*(O/W/32/O) | 1 | 0.01444 | 2.118 | 0.03 | * |
| *tet*X | 1 | 0.01429 | 2.0961 | 0.043 | * |
| Residual | 62 | 0.42267 |  |  |  |
| 7.3. Testing the significance of CCA axes | | | |  |  |
| CCA1 | 1 | 0.19507 | 28.6141 | 0.001 | *** |
| CCA2 | 1 | 0.15807 | 23.1872 | 0.001 | *** |
| CCA3 | 1 | 0.10889 | 15.9727 | 0.001 | *** |
| **8. A CCA model of other resistance genes** | | | |  |  |
| 8.1. Testing the significance of the CCA model | | | |  |  |
| Model | 48 | 0.792696419 | 2.158175418 | 0.001 |  |
| Residual | 47 | 0.359647276 | NA | NA |  |
| 8.2. Testing the significance of terms (environmental variables) | | | |  |  |
| *arn*C | 1 | 0.07998 | 10.4523 | 0.001 | *** |
| *DHFR* | 1 | 0.05797 | 7.5756 | 0.001 | *** |
| *nim*C | 1 | 0.05107 | 6.6739 | 0.001 | *** |
| *nim*J | 1 | 0.03498 | 4.5707 | 0.001 | *** |
| *dhfr*Ib | 1 | 0.03269 | 4.2717 | 0.004 | ** |
| *dhfr*XII | 1 | 0.0216 | 2.8227 | 0.005 | ** |
| *mcr* | 1 | 0.02525 | 3.2993 | 0.004 | ** |
| *pat*A | 1 | 0.02319 | 3.0304 | 0.006 | ** |
| *dfr*A1 | 1 | 0.01688 | 2.2054 | 0.047 | * |
| *inu*A | 1 | 0.02043 | 2.6692 | 0.016 | * |
| *mcr* | 1 | 0.02179 | 2.8471 | 0.019 | * |
| *nov*A | 1 | 0.01868 | 2.4415 | 0.025 | * |
| *ugd* | 1 | 0.0239 | 3.1232 | 0.016 | * |
| *van*D | 1 | 0.0217 | 2.8363 | 0.02 | * |
| Residual | 47 | 0.35965 |  |  |  |
| 8.3. Testing the significance of CCA axes | | | |  |  |
| CCA1 | 1 | 0.21448 | 36.3787 | 0.001 | *** |
| CCA2 | 1 | 0.14609 | 24.7782 | 0.001 | *** |
| CCA3 | 1 | 0.11464 | 19.4437 | 0.002 | ** |
|  |  |  |  |  |  |
| * Pr(>F) <0.05, ** Pr(>F) <0.01, *** Pr(>F) <0.001 | | | |  |  |

**Fig. S1.** Box-and-whisker plot with individual data points for Shannon diversity index values of piglets at day 3 pre-weaning (D.−3), weaning day (D.0), day 3 post-weaning (D.3) and day 8 post-weaning (D.8).


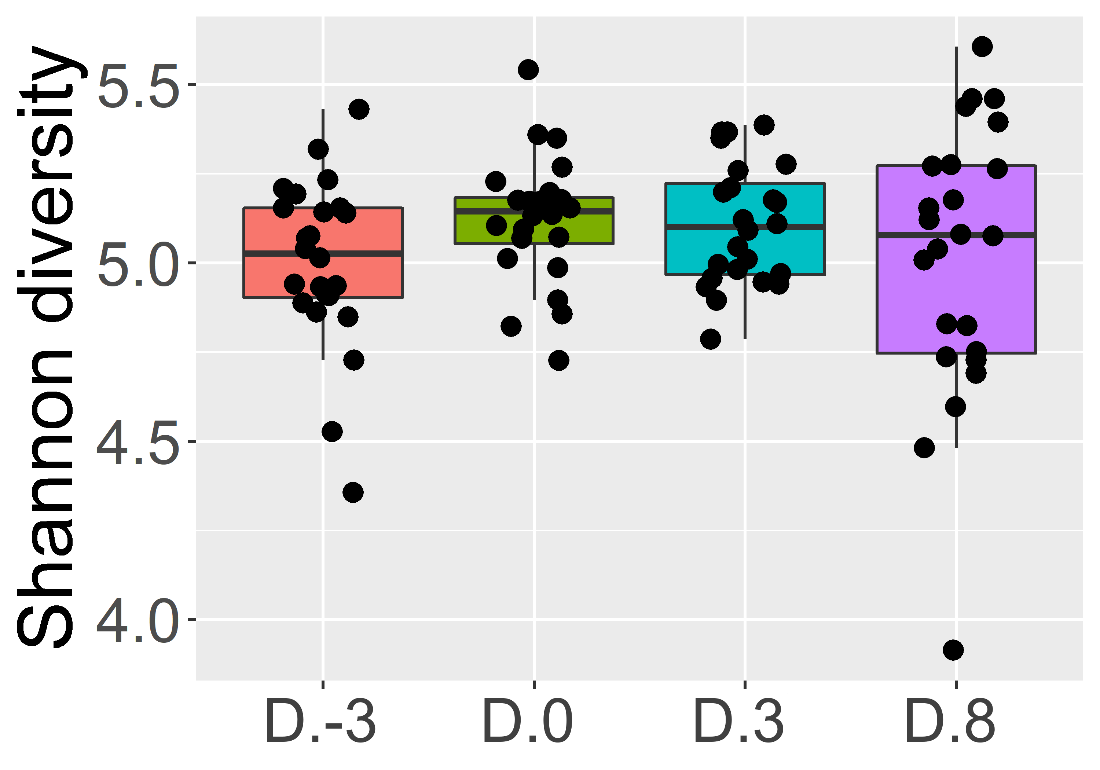

Supplement: Supplementary file 1 — Supplementary Information. [file 41598_2021_97586_MOESM1_ESM.docx]
